# Gender differences in screening for glucose perturbations, cardiovascular risk factor management and prognosis in patients with dysglycaemia and coronary artery disease: results from the ESC-EORP EUROASPIRE surveys

**DOI:** 10.1186/s12933-021-01233-6

**Published:** 2021-02-11

**Authors:** Giulia Ferrannini, Dirk De Bacquer, Pieter Vynckier, Guy De Backer, Viveca Gyberg, Kornelia Kotseva, Linda Mellbin, Anna Norhammar, Jaakko Tuomilehto, David Wood, Lars Rydén, EUROASPIRE IV & V Investigators

**Affiliations:** 1 Cardiology Unit, Department of Medicine K2, Karolinska Institutet, Solnavägen 1, 17177 Stockholm, Sweden; 2 Department of Public Health and Primary Care, Ghent University, C. Heymanslaan 10, 9000 Ghent, Belgium; 3 Department of Neurobiology, Centre for Family Medicine, Care Sciences and Society, Karolinska Institutet, Alfred Nobels allé 23, D2, 141 83 Huddinge, Sweden; 4 National Institute for Prevention and Cardiovascular Health, National University of Ireland-Galway, University Road, Galway, H91 TK33 Republic of Ireland; 5 St Mary’s Hospital, Imperial College Healthcare NHS Trust, The Bays, S Wharf Rd, Paddington, London, W2 1NY UK; 6 Heart, Vascular and Neuro Theme, Karolinska University Hospital, Eugeniavägen 3, 17164 Stockholm, Sweden; 7 Capio St Görans Hospital, Sankt Göransplan 1, 11219 Stockholm, Sweden; 8 Public Health Promotion Unit, Finnish Institute for Health and Welfare, Mannerheimintie 166, 00271 Helsinki, Finland; 9 Department of Public Health, University of Helsinki, Helsinki, Finland; 10 Diabetes Research Group, King Abdulaziz University, Jeddah, 21589 Saudi Arabia

**Keywords:** Diabetes, Coronary artery disease, Gender, Impaired glucose tolerance, Prevention

## Abstract

**Background:**

Gender disparities in the management of dysglycaemia, defined as either impaired glucose tolerance (IGT) or type 2 diabetes (T2DM), in coronary artery disease (CAD) patients are a medical challenge. Recent data from two nationwide cohorts of patients suggested no gender difference as regards the risk for diabetes-related CV complications but indicated the presence of a gender disparity in risk factor management. The aim of this study was to investigate gender differences in screening for dysglycaemia, cardiovascular risk factor management and prognosis in dysglycemic CAD patients.

**Methods:**

The study population (n = 16,259; 4077 women) included 7998 patients from the ESC-EORP EUROASPIRE IV (EAIV: 2012–2013, 79 centres in 24 countries) and 8261 patients from the ESC-EORP EUROASPIRE V (EAV: 2016–2017, 131 centres in 27 countries) cross-sectional surveys. In each centre, patients were investigated with standardised methods by centrally trained staff and those without known diabetes were offered an oral glucose tolerance test (OGTT). The first of CV death or hospitalisation for non-fatal myocardial infarction, stroke, heart failure or revascularization served as endpoint. Median follow-up time was 1.7 years. The association between gender and time to the occurrence of the endpoint was evaluated using Cox survival modelling, adjusting for age.

**Results:**

Known diabetes was more common among women (32.9%) than men (28.4%, p < 0.0001). OGTT (n = 8655) disclosed IGT in 17.2% of women vs. 15.1% of men (p = 0.004) and diabetes in 13.4% of women vs. 14.6% of men (p = 0.078). In both known diabetes and newly detected dysglycaemia groups, women were older, with higher proportions of hypertension, dyslipidaemia and obesity. HbA1c was higher in women with known diabetes. Recommended targets of physical activity, blood pressure and cholesterol were achieved by significantly lower proportions of women than men. Women with known diabetes had higher risk for the endpoint than men (age-adjusted HR 1.22; 95% CI 1.04–1.43).

**Conclusions:**

Guideline-recommended risk factor control is poorer in dysglycemic women than men. This may contribute to the worse prognosis in CAD women with known diabetes.

## Background

Dysglycaemia, defined as the presence of either impaired glucose tolerance (IGT) or type 2 diabetes (T2DM), represents a major cause of morbidity and mortality worldwide, mainly due to its high risk of vascular complications, including coronary artery disease (CAD) [1, 2]. International guidelines recommend both screening for glucose perturbations and a comprehensive cardiovascular (CV) risk factor control in people with dysglycaemia and CAD [3]. However, real-world data show that guideline adherence is poor and in need of considerable improvement [4, 5].

The presence of gender disparities in the management of dysglycaemia in CAD patients is a serious medical challenge [6–8]. First of all, despite men averagely receiving a diagnosis of dysglycaemia at a younger age, cardiometabolic risk factors seem to escalate to a greater extent in women as they proceed from normo- to dysglycaemia [9–11]. Secondly, although male sex is associated with a higher absolute risk of CAD several studies reported on an excess relative risk of diabetes-associated CAD in women [8, 11–13]. Historically, this has been considered to be due to both the attenuating effect of diabetes on the cardiometabolic protection conferred by oestrogens [14] and to the fact that that women, being treated less effectively than men, are less likely to achieve recommended risk factor targets [15, 16]. More recent data from two nationwide cohorts of patients with atherosclerotic disease manifestations partially revisited these assumptions, suggesting no difference between women and men as regards the risk for diabetes-related CV complications, but confirming the existence of a gender disparity in risk factor management [17, 18]. The results of such studies are, however, partly conflicting and may be diverse among various populations [19]. One reason is that these observations were made based on registry-derived information i.e. not on standardised investigations, which in itself may cause bias, and another that these populations were domestic.

The main objective of the present study is to investigate gender differences in screening for dysglycaemia, CV risk factor management in a large homogeneous cohort of dysglycemic patients with verified CAD subjected to a standardised examination within the framework of the European Action on Secondary and Primary Prevention by Intervention to Reduce Events (EUROASPIRE) IV and V [20, 21]. A secondary objective was to report CV outcomes in relation to gender.

## Methods

### Study population

The study population (n = 16,259; 4077 women) consists of 7998 patients from the ESC-EORP EUROASPIRE IV (EAIV: 2012–2013, 79 centres in 24 countries) and 8261 patients from the ESC-EORP EUROASPIRE V (EAV: 2016–2017, 131 centres in 27 countries) cohorts. Details on the participating countries and centres are presented as appended information (see Additional file 1: Appendix S1).

Consecutively diagnosed CAD patients, 18 to 80 years old, were identified from diagnostic registers, hospital discharge lists or other sources. They had to be hospitalised for an elective or emergency coronary artery bypass grafting (CABG), an elective or emergency percutaneous coronary intervention (PCI), an acute myocardial infarction (MI) (ICD-10 I21) or acute myocardial ischemia (ICD-10 I20) three (EUROASPIRE IV) or two years (EUROASPIRE V) to six months prior to the date of the study visit. Extensive information was collected by means of standardized interviews and investigations by centrally trained research staff, using standardized methods and with uniform equipment. Data were electronically submitted to the data management centre (EURObservational Research Programme (EORP), ESC, Sophia-Antipolis, France).

### Methods

Major efforts were implemented in standardizing all measurements used in the participating centres, including central training of observers.

*Height* (cm) and *weight* (kg) were recorded in light indoor clothes without shoes (Scales 701 and Measuring stick model 220; SECA Medical Measuring Systems and Scales, Birmingham, U.K.).

*Waist circumference* was measured with the patient standing, using a metal tape applied horizontally at the point midway in the midaxillary line between the lowest rim of the ribcage and the superior iliac crest.

*Blood pressure* Systolic and diastolic blood pressure was measured twice on the right upper arm in the sitting position using an automatic digital sphygmomanometer (Omron M6; OMRON Corporation, Kyoto, Japan). The mean of both measurements was used for the analyses.

*Laboratory investigations* Venous blood was drawn after $\ge$ 10 h of fasting for measuring serum total and high density lipoprotein-cholesterol (HDL-C), triglycerides, and glycated haemoglobin A1c (HbA1c) while low density cholesterol- cholesterol (LDL-C) was calculated by Friedewald’s formula [22]. Samples were stored locally at − 70 °C and subsequently sent to a central laboratory for final storage and analyses (Disease Risk Unit, National Institute for Health and Welfare, Helsinki, Finland) accredited by the Finnish Accreditation Service, fulfilling the requirements of the standard SFS-EN International Organization for Standardization/International Electrotechnical Commission 17,025:2005. Total and HDL-C and triglycerides were analysed on a clinical chemistry analyser (Abbot Architect Analyzer; Abbott Laboratories, Abbott Park, IL) using an enzymatic method for measuring total cholesterol. HbA1c was measured with an immunoturbidimetric International Federation of Clinical Chemistry and Laboratory Medicine aligned method (Abbot Architect Analyzer) in fasting venous whole blood sampled in an EDTA tube.

All patients without known diabetes were offered an Oral Glucose Tolerance test (OGTT; 75 g glucose in 200 mL water). Plasma glucose (PG) was analysed locally in the fasting state (FPG) and 2 h after the glucose load (2 h-PG) with a photometric point-of-care technique (Glucose 201 + (EAIV) or Glucose 201RT (EAV); HemoCue, Ängelholm, Sweden) [23]. Since the HemoCue technique is cholesterol-sensitive, glucose values were corrected for cholesterol according to the formula: HemoCue glucose + 0.15 x (total cholesterol − 5). HemoCue automatically converts the venous blood glucose to plasma glucose by using the International Federation of Clinical Chemistry and Laboratory Medicine (IFCC) recommendation: plasma glucose = 1.11 × whole blood glucose [24].

*Pharmacological treatment:* Information on medication intake was based on the self-reported use at the time of the interview.

### Definitions

*Educational level* was defined as “low” if the patient reported no further education than completed primary school.

*Smoking* was defined as self-reported smoking and/or a breath carbon monoxide higher than 10 ppm by means of Smokerlyzer (Bedfont Scientific, Model Micro1) at the time of interview. *Persistent smoking* was defined as smoking at the time of interview among those who smoked the month prior to the index event.

*Overweight* was defined as a Body Mass Index (BMI) between 25 and 29.9 kg/m^2^ and *obesity* as BMI ≥ 30 kg/m^2^. *Central obesity* was defined as a waist circumference ≥ 88 cm for women and ≥ 102 cm for men.

The *physical activity target* was defined by the question: “Do you take regular physical activity for at least 30 min on average five times a week?’’.

The *use of four cardioprotective drugs*, consisting of antiplatelet drugs, beta-blockers, renin–angiotensin–aldosterone system (RAAS) blockers, and lipid-lowering drugs was assessed at the interview visit.

*Treatment target attainment* was assessed for blood pressure and LDL-C according to the 2012 European Guidelines on Cardiovascular Disease Prevention in clinical practice [25] and the 2013 European Guidelines for Diabetes, Pre-Diabetes and Cardiovascular Disease [26].

*Glycaemic state* was defined according to World Health Organization as outlined in Additional file 1: Table S1 [27].

*Previously known diabetes* is defined as a self-reported history of diabetes or use of any glucose-lowering medication.

*Newly detected dysglycaemia* is defined as the presence of IGT or T2DM according to the OGTT, performed in patients without previously known diabetes.

*Anxiety and depression scores* were estimated by means of the Hospital Anxiety and Depression Scale (HADS) questionnaire [28].

*Generic health status* was assessed by means of VAS-scale of the EuroQoL 5D questionnaire, varying from 0 (the worst possible health status) to 100 (the best possible health status) [29].

### Follow-up

All centres were asked to complete a follow-up questionnaire for the EUROASPIRE IV and V participants. To be eligible for the follow-up part the retrieved information had to cover ≥ 12 months follow-up on ≥ 90% of the patients from the participating centres. Fatal events were recorded as death from the following causes: CAD, stroke, other vascular, cancer, other causes and unknown. Nonfatal events were recorded as hospitalisation for PCI, CABG, acute MI, stroke/transient ischemic attack, and heart failure. Follow-up information was obtained from patient interviews, medical records, or external registries or databases (mortality registries, local records and archives) or, if needed, by contacting relatives or a family physician.

The first of CV death or hospitalisation for any of the following non-fatal events: MI, non-fatal stroke, heart failure, CABG or PCI served as the endpoint, and time at risk for developing the endpoint was calculated from the baseline study visit. In the absence of an event, time at risk was censored at the last date of follow-up.

### Ethics

Local Ethics Committees approval was obtained via the National Coordinators for each participating country in both surveys. Signed informed consent was obtained from each participant and stored locally in the patient file.

### Statistical analysis

Distributions of baseline characteristics were summarized according to means, standard deviations and proportions. Characteristics of women and men, both in the previously known T2DM group and in the newly detected dysglycaemia group, were compared by using the Mann–Whitney U-test for continuous variables and Fisher’s exact test for categorical variables. The association between gender and time to the occurrence of the endpoint was evaluated using Cox survival modelling, adjusting for age. The assumption of proportionality of hazards in women and men in time, was checked by fitting a gender-by-time interaction term in the model. A double-sided type I error level of α = 0.05 was used to indicate statistical significance. All data analyses were undertaken using SAS statistical software (release 9.4) at the Department of Public Health and Primary Care, Ghent University, Belgium.

### Role of the founding source

The EUROASPIRE IV and V surveys were performed under the auspices of the European Society of Cardiology, EURObservational Research Programme. The sponsors of the EUROASPIRE surveys (detailed in the Funding section) had no role in the design, data collection, data analysis, data interpretation, decision to publish, or writing the manuscript.

## Results

**Fig. 1:**
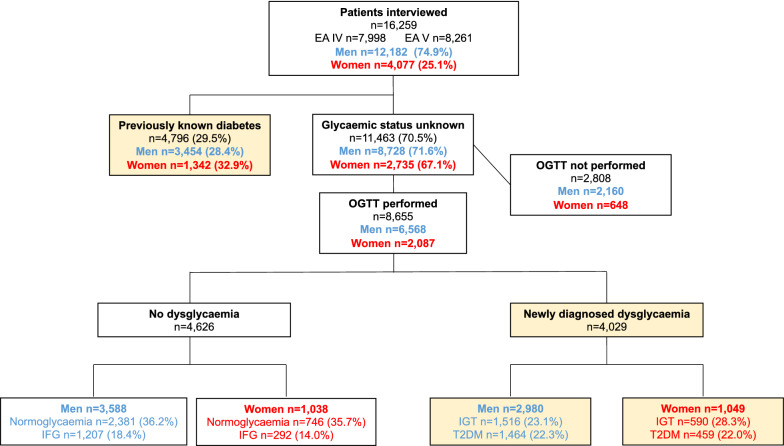
Flowchart of the patients and their glycaemic classification according to the oral glucose tolerance test (OGTT). The dysglycemic study population is highlighted with yellow background. *EA* EUROASPIRE, *IFG* impaired fasting glucose, *IGT* impaired glucose tolerance, *T2DM* type 2 diabetes mellitus

Of the 16,259 patients 4077 (25.1%) were women (Fig. 1). A total of 4796 (29.5%) had previously known diabetes (women 32.9%; men 28.4%; p < 0.0001), whereof 97% T2DM. An OGTT was performed in 8655 (80%) of the remaining 11,463 patients. The proportion of women and men who did not undergo such screening did not differ (p = 0.26). The final study population of dysglycemic individuals with CAD comprised 4796 patients with previously known T2DM and 4029 with newly detected dysglycaemia.

### Glycaemic state and screening for glucose perturbations

**Fig. 2:**
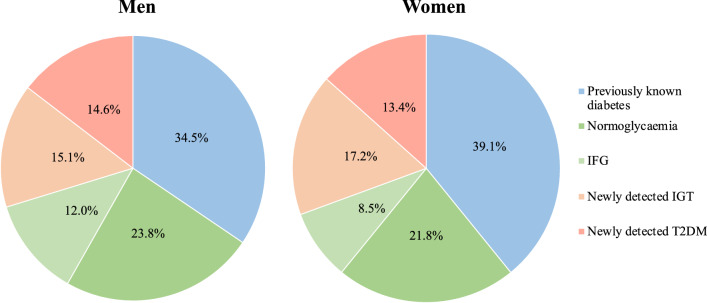
The distribution of glycaemic state divided by gender in the study population. *IFG* impaired fasting glucose, *IGT* impaired glucose tolerance, *T2DM* type 2 diabetes mellitus

In the study population whose glycaemic status was known, equal proportions of women (21.8%) and men (23.8%; p = 0.55 after age adjustment) were normoglycaemic, while the proportion of IGT was significantly higher among women (17.2%) than men (15.1%; p = 0.015) and that of impaired fasting glucose (IFG) lower in women than men (p < 0.0001) (Fig. 2). Slightly more men were newly diagnosed with T2DM (women 13.4% vs. men 14.6%; p = 0.020).

**Fig. 3:**
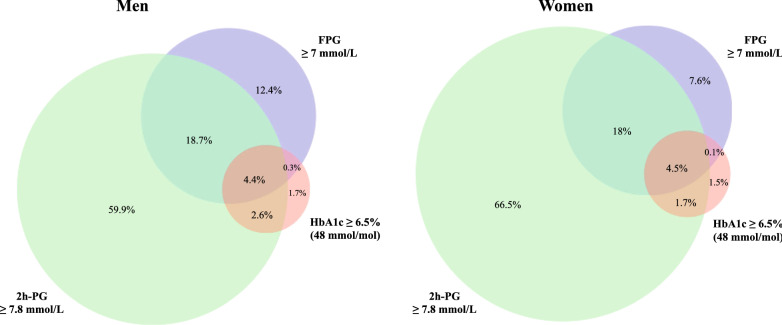
Proportions and their overlap between screening with different methods [FPG ≥ 7 mmol/L, 2hPG mmol/L ≥ 7.8 mmol/L, HbA1c $\ge$ 6.5% (48 mmol/mol)] and their combinations in men and women with newly detected dysglycaemia (IGT or T2DM). *2 h-PG* 2-h post-load glucose, *FPG* fasting plasma glucose, *HbA1c* glycated haemoglobin, *IGT* impaired glucose tolerance, *T2DM* type 2 diabetes mellitus

Screening for dysglycaemia based on FPG, 2 h-PG and HbA1c values (alone or in combination) showed that more women (66.5%) than men (59.9%) (p < 0.0001) would not have been identified as dysglycemic without the 2 h-PG value (Fig. 3). Only 4.5% of women and 4.4% of men were identified as dysglycaemic simultaneously by all three tests.

### *Clinical characteristics* (Table 1)

**Table 1:** Baseline characteristics according to gender and glycaemic category

	Previously known diabetes			Newly detected dysglycaemia			
	Women (n = 1342)	Men (n = 3454)	P-value	Women (n = 1049)	Men (n = 2980)		P-value^a^
Age (years)	66.6 (8.7)	64.6 (8.9)	< *0.0001*	66.8 (8.9)	64.0 (9.6)		< *0.0001*
Low educational level	24.5 (324/1323)	16.1 (548/3401)	< *0.0001*	19.9 (208/1046)	15.4 (456/2960)		*0.0010*
Currently smoking	16.1 (216/1342)	29.0 (1002/3454)	< *0.0001*	22.1 (232/1049)	33.1 (987/2980)		< *0.0001*
Persistent smoking	53.7 (116/216)	53.0 (531/1002)	0.88	48.3 (112/232)	44.9 (443/987)		0.38
Physical activity level on target	45.8 (594/1297)	55.2 (1852/3358)	< *0.0001*	51.5 (528/1026)	57.0 (1665/2922)		*0.0024*
Medical history							
Hypertension	87.2 (1158/1328)	79.5 (2712/3410)	< *0.0001*	80.8 (841/1041)	73.7 (2172/2947)		< *0.0001*
Dyslipidaemia	78.4 (1011/1289)	75.0 (2496/3327)	*0.016*	75.0 (766/1021)	68.9 (1977/2869)		*0.00023*
Anthropometrics—vitals							
BMI (kg/m^2^)	31.6 (5.9)	30.1 (4.8)	*0.0001*	29.8 (5.5)	29.0 (4.3)		*0.0023*
Obesity	57.7 (754/1306)	45.6 (1550/3396)	*0.0001*	43.9 (460/1048)	36.6 (1089/2977)		< *0.0001*
Central obesity	86.7 (1080/1246)	63.6 (2077/3266)	< *0.0001*	77.3 (789/1021)	54.2 (1577/2909)		< *0.0001*
SBP (mmHg)	138.3 (20.3)	137.2 (18.9)	0.18	134.4 (19.5)	134.2 (18.6)		0.99
DBP (mmHg)	79.3 (11.6)	80.1 (11.0)	*0.036*	79.3 (11.3)	80.3 (11.0)		*0.0011*
Heart rate (bpm)	70.9 (11.2)	68.8 (11.1)	< 0.0001	69.1 (11.1)	67.9 (11.4)		*0.0018*
Laboratory central assessment							
LDL-C (mmol/L)	2.52 (1.04)	2.21 (0.90)	< *0.0001*	2.76 (1.08)	2.46 (0.91)		< *0.0001*
HDL-C (mmol/L)	1.15 (0.31)	1.03 (0.26)	< *0.0001*	1.29 (0.31)	1.12 (0.26)		< *0.0001*
Total C (mmol/L)	4.56 (1.38)	4.08 (1.14)	< *0.0001*	4.74 (1.22)	4.31 (1.10)		< *0.0001*
Triglycerides (mmol/L)	1.99 (1.66)	1.91 (1.56)	*0.0005*	1.55 (0.81)	1.62 (1.05)		0.83
FPG (mmol/L)	n.a	n.a	n.a	6.53 (0.97)	6.63 (1.08)		*0.0072*
2 h-PG (mmol/L)	n.a	n.a	n.a	9.81 (2.37)	9.65 (2.51)		0.070
HbA1c (NGSP, %)—(IFCC, mmol/mol)^b^	7.36 (1.70)—57	7.07 (1.49)—54	< *0.0001*	5.80 (0.46)—40	5.79 (0.56)—40		*0.014*
Creatinine (µmol/L)	85.3 (53.7)	97.9 (53.2)	< *0.0001*	78.0 (28.5)	93.4 (37.5)		< *0.0001*
eGFR (mL/min/1.73 m^2^)	89.2 (24.5)	76.9 (21.2)	< *0.0001*	93.9 (20.2)	78.5 (18.1)		< *0.0001*
Pharmacological treatment							
RAAS blockers	80.5 (1070/1329)	79.7 (2723/3418)	0.55	72.8 (759/1042)	76.0 (2251/2963)		0.046
Beta blockers	85.7 (1138/1328)	83.7 (2860/3419)	0.084	83.3 (869/1043)	82.5 (2444/2964)		0.54
Antiaggregants	93.7 (1242/1325)	93.1 (3184/3419)	0.48	91.3 (952/1043)	92.6 (2747/2965)		0.16
Lipid lowering	83.7 (1112/1329)	87.6 (2991/3414)	0.00046	83.2 (866/1041)	85.7 (2540/2963)		0.055
Statins	82.5 (1097/1329)	86.5 (2952/3414)	*0.00071*	82.1 (855/1041)	85.0 (2519/2963)		*0.030*
Ezetimibe	3.1 (41/1329)	3.1 (105/3419)	1.00	2.2 (23/1043)	2.5 (75/2966)		0.64
All four above	58.6 (775/1323)	58.9 (2009/3413)	0.87	52.0 (541/1040)	55.0 (1628/2960)		0.10
Diuretics	48.2 (640/1329)	41.5 (1420/3418)	< *0.0001*	37.2 (388/1042)	29.3 (869/2964)		< *0.0001*
Glucose-lowering drugs	92.7 (1244/1342)	91.6 (3162/3451)	0.24	n.a	n.a		n.a
Antidepressant/antianxiety drugs	9.6 (128/1330)	7.1 (224/3418)	*0.004*	9.8 (102/1042)	5.2 (155/2963)		< *0.0001*
Advice on lifestyle changes							
Stop smoking	83.7 (169/202)	86.2 (833/966)	0.38	84.9 (191/225)	84.5 (792/937)		1.00
Healthy diet	91.2 (1105/1212)	89.4 (2849/3186)	0.093	83.6 (806/964)	85.8 (2347/2734)		0.10
Weight loss	72.8 (929/1276)	74.0 (2463/3327)	0.41	63.9 (647/1012)	67.4 (1953/2898)		*0.049*
Increase physical activity	59.7 (762/1277)	65.6 (2176/3315)	*0.00018*	58.8 (597/1016)	63.3 (1821/2879)		*0.012*
Actions taken to change lifestyle							
Stop smoking	79.7 (165/207)	76.0 (728/958)	0.28	85.8 (193/225)	82.0 (769/938)		0.20
Healthy diet	89.9 (1064/1183)	89.1 (2809/3152)	0.47	90.1 (858/952)	89.0 (2385/2679)		0.36
Weight loss	58.8 (741/1260)	61.3 (2021/3299)	0.14	58.0 (583/1006)	60.1 (1720/2861)		0.23
Increase physical activity	25.0 (301/1203)	33.5 (1065/3183)	< *0.0001*	32.3 (307/ 951)	40.2 (1100/2738)		< *0.0001*
Attended a CPRP	27.0 (355/1315)	33.7 (1147/3404)	< *0.0001*	32.1 (332/1035)	35.7 (1052/2943)		*0.034*
Attended a DEP	25.8 (314/1217)	26.0 (809/3116)	0.94	n.a	n.a		n.a
Quality of life assessment							
EQ-5D VAS score	56.1 (26.3)	60.6 (27.5)	< *0.0001*	59.2 (27.1)	63.4 (27.7)		< *0.0001*
HeartQoL Global score	1.79 (0.72)	2.14 (0.67)	< *0.0001*	1.97 (0.70)	2.27 (0.62)		< *0.0001*
HeartQoL Physical score	1.81 (0.74)	2.17 (0.70)	< *0.0001*	2.00 (0.72)	2.31 (0.64)		< *0.0001*
HeartQoL Emotional score	1.73 (0.78)	2.08 (0.72)	< *0.0001*	1.91 (0.77)	2.18 (0.69)		< *0.0001*

Cell entries are % (n/total number) or mean (SD)

Italic values indicate statistically significant p-values

*2 h-PG* 2-h post-load glucose, *BMI* Body Mass Index, *CPRP* Cardiac Prevention and Rehabilitation Programme, *DBP* diastolic blood pressure, *DEP* diabetes educational programme, *eGFR* estimated glomerular filtration rate, *EQ-5D* EuroQol 5D Questionnaire, *FG* fasting plasma glucose, *HbA1c* glycated haemoglobin, *HDL-C* high density lipoprotein cholesterol, *Heart QoL* Heart Quality of Life, *IFCC* International Federation of Clinical Chemistry, *LDL-C* low-density lipoprotein cholesterol, *NGSP* National Glycohemoglobin Standardization Program, *RAAS* renin–angiotensin–aldosterone system, *SBP* systolic blood pressure, *T2DM* type 2 diabetes mellitus, *Total C* total cholesterol, *VAS* visual analogue scale

^a^significance level for testing differences between men and women in each diagnostic group

^b^mean HbA1c levels were converted using the NGSP calculator at http://www.ngsp.org/convert1.asp

Women were older than men, had a lower educational level, and had a higher frequency of hypertension, dyslipidaemia and obesity in both glycaemic categories.

Renal function, expressed as eGFR, was better in women than in men. Total cholesterol and LDL-C levels were significantly higher (p < 0.0001) in women than in men, both in patients with previously known T2DM and among those with newly detected dysglycaemia. Serum triglycerides were significantly higher in women than in men in those with previously known T2DM. The glycaemic control as assessed by HbA1c was less strict in women than in men with known T2DM (p < 0.0001) (Table 1); in women HbA1c < 7% (53 mmol/mol) was 51.0% and in men 57.3% (p < 0.0001).

Microvascular complications were significantly more common in women than among men with previously known T2DM: retinopathy (25.0% vs. 15.8%; p < 0.0001), renal involvement (4.8% vs. 3.2%; p = 0.03) and neuropathy (23.5% vs. 14.9%; p < 0.0001).

### *Lifestyle habits* (Table 1)

Less women than men were current smokers, but the proportion of persistent smokers (patients who were smoking at the time of the recruiting event and still smoking at interview) was similar in both genders across the two glycaemic categories. Less women than men had been advised on and increased their physical activity. Significantly less women than men attended a Cardiac Prevention and Rehabilitation Programme in both glycaemic categories, but there was no gender difference in the attendance at a Diabetes Educational Programme in patients with previously known T2DM. The scores expressing quality of life, i.e., EuroQoL 5D and HeartQoL, were significantly lower in women than men. Women in both glycaemic categories were prescribed significantly more antidepressant/antianxiety drugs than men.

### Pharmacological treatment and targets attainment

The proportion of patients taking each of four cardioprotective drug classes and their combination did not differ according to gender in the two glycaemic categories, with the exception of RAAS blockers that were prescribed less frequently to women than men with newly detected dysglycaemia (72.8% vs. 76.0%; p = 0.046) and lipid-lowering therapy prescribed less frequently to women than men with previously known T2DM (83.7% vs. 87.6%; p = 0.00046) (Table 1). The combination of all four cardioprotective drugs was prescribed to < 60% of patients, with no significant differences between genders. Compared with men, women with known T2DM were more frequently prescribed insulin (33% vs. 25.2%; p < 0.0001) while smaller proportions of women used metformin (53.2% vs. 58.9%; p < 0.001).

**Fig. 4:**
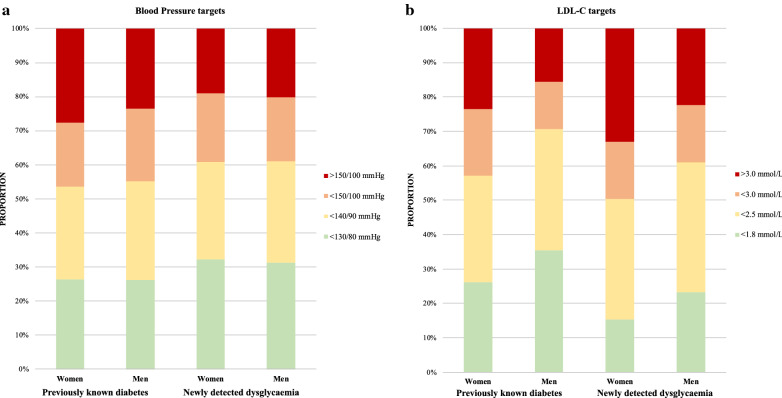
Proportion of patients with previously known T2DM and newly detected dysglycaemia reaching different blood pressure (**a**) and LDL-C (**b**) targets in the total cohort

The proportion of men and women reaching different blood pressure and LDL-C targets is shown in Fig. 4. Among patients with previously known T2DM more women than men had blood pressure $\ge$ 150/100 mmHg (27.7% vs. 23.5%; p < 0.0034) and LDL-C $\ge$ 3.0 mmol/L (23.6% vs. 15.6%; p < 0.0001) whereas they achieved an LDL-C level < 1.8 mmol/L in a significantly lower proportion (26.1%; vs. men 35.5%; p < 0.0001). A similar pattern was observed in patients with newly diagnosed dysglycaemia with women having a higher proportion of LDL-C ≥ 3.0 mmol/L and a lower proportion of LDL-C < 1.8 mmol/L.

### CV events during follow-up

The median follow-up time was 1.7 years, and the number of events in patients with newly detected dysglycaemia were 105 in women and 340 in men. The corresponding numbers in patients with known T2DM were 233 and 500 respectively. A detailed description of the events is given in Additional file 1: Table S2. The total number of observed person-years was 23,703. The age-adjusted incidence of the study endpoint was significantly higher in women than in men with known T2DM (125.4 vs. 100.8/1000 person-years) with a hazard ratio (95% confidence interval (CI); p-value) of women vs. men of 1.22 (1.04–1.43; p = 0.015). There was no significant gender difference in the age-adjusted incidence of the endpoint in patients with newly detected dysglycaemia (women vs. men: incidence 65.9 vs. 75.4/1000 person-years), with a hazard ratio of 0.86 (95% CI 0.69–1.08; p = 0.19).

## Discussion

The main finding of the present study is that women with CAD and dysglycaemia, compared with men, are more burdened with CV risk factors, less often participate in cardiac rehabilitation, report less physical activity and less often achieve satisfactory risk factor control—all important factors for preventing further glucose deterioration and future CV events. Other important findings are the significantly higher prevalence of microvascular complications seen among women and that a majority of women (67%) and large part of men (60%) would not have been identified as dysglycaemic without an OGTT.

The EUROASPIRE surveys were developed to determine how the European guidelines on CV disease prevention are implemented in a real-world setting. The Guidelines on Diabetes, Pre-diabetes and Cardiovascular Diseases, first issued in 2003 and recently updated in 2019, reinforced the importance of a multi-targeted approach to CV risk factors [3]. There is a substantial need for a general improvement in detection and treatment of dysglycaemia [4]. Attention to glycaemic perturbations in women has been advocated in the gynaecologic field with regard to the polycystic ovary syndrome, premature menopause, gestational diabetes and pre-eclampsia [7]. The present study underlines the importance of widening this attention to women with dysglycaemia and CAD, most of them post-menopausal.

### Screening for glucose perturbations

Appropriate screening for glucose metabolism perturbations is of particular importance in women, since it appears that the advantage given by their later CAD presentation compared with men is eliminated by the presence of diabetes [7, 13]. The DECODE (Diabetes Epidemiology: Collaborative analysis Of Diagnostic criteria in Europe) study showed that the hazard ratios for CV mortality was higher in women than men with newly diagnosed diabetes compared with their normoglycaemic counterparts [30]. Indeed, in our population of patients burdened by CAD, a significantly higher proportion of women had previously known T2DM, in keeping with the Euro Heart Survey in 2007 [8]. Further, more women than men were identified as dysglycemic due to a higher proportion of IGT, as in the population-based DECODE study [30]. Importantly, IGT is a pre-diabetes state prognostically as unfavourable as newly detected T2DM, and only detectable by means of an OGTT [31, 32]. The higher prevalence of IGT in women with established CAD compared with men has not been previously reported. This finding underlines the importance of using the OGTT in both women and men with CAD, rather than relying on a fasting glucose only, unless it already indicates the presence of diabetes (≥ 7.0 mmol/l) and understanding that HbA1c is an even less reliable screening test [31].

### Pharmacological and lifestyle management of CV risk factors

In order to avoid conclusions based on blood pressure and LDL-C levels just above the recommended treatment targets, we presented proportions at different levels (see Fig. 4).

Even taking these less strict targets into consideration, target attainment remains poor. Considering that contemporary guidelines recommend even stricter cut-offs for blood pressure (< 130/80 mmHg) and LDL-C (< 1.4 mmol/L) in all CAD patients [3] than those recommended at the time for EUROASPIRE IV and V, the present findings must be considered markedly disadvantageous for the future prognosis of women with CAD and dysglycaemia. A German population study showed that women with T2DM and CVD were more likely to have blood pressure, LDL-C and HbA1c uncontrolled compared with men [16], as was also reported from more than 8000 Croatian T2DM patients [33]. The SWEDEHEART registry has consistently reported that less women than men reached the blood pressure and LDL-C targets one year after an acute MI [34]. This seems to apply to ethnically and socioeconomically heterogeneous cohorts, as a study on CAD patients (32% T2DM) conducted in 11 countries reported that all pharmacological and lifestyle targets were achieved by a significantly lower proportion of women than men [35].

HbA1c in women with previously known T2DM was higher than in men, despite glucose-lowering drugs being prescribed in similar proportions [33]. It has been speculated that females with T2DM are more exposed to metabolic disturbances than males [7, 36], but this does not offset the fact that the risk factor intervention is less well implemented among the women.

The only favorable lifestyle-related aspect for women was that they smoked less than men. Otherwise, women achieved the desired physical activity target and attended cardiac prevention and rehabilitation programs in lower proportions than men. That their quality of life was lower and that they were more often prescribed antidepressants and antianxiety drugs compared with men is in accordance with the finding that a cluster of CV risk factors including low levels of physical activity, anxiety, depression and unhealthy lifestyles was more common in women [37]. Moreover, despite being advised on and having pursued weight loss in similar proportions, obesity remained significantly more prevalent among women than men, especially in those with previously known T2DM, in whom the central obesity was very common (86.7%). Finally, as discussed in a recent editorial, women and men might have different preferences, compliance and response to lifestyle management, an area in need of further research [38].

### CV outcomes

The finding of a poorer prognosis in CAD women with known T2DM is in line with their less well managed risk factor control. A recent Danish study reported that the relative rates of CV complications associated with T2DM were higher in women than men in all ages [18] and also seen in a large British study where only incident cases, i.e. patients with recently diagnosed T2DM, were included [17]. Although there may be analytical discrepancies to account for when discussing these studies, the main message seems to be that women with dysglycaemia are more exposed to CV complications than men, not the least since they are less well treated. Thus, early screening of CAD women in order to detect dysglycaemia at an early stage and with sensitive methods should be encouraged. This is particularly important considering their higher prevalence of microvascular complications, which at least partially may explain their worse outcome. That microvascular disease is more prevalent in post-menopausal women than men underlines the need to address this in risk stratification of CAD patients by non-invasive measures such as spot albuminuria and cardiac autonomic neuropathy, in addition to OGTT [11, 39–41].

### Strengths and limitations

Our study has several strengths. First, the EUROASPIRE surveys report data from a large cohort of dysglycemic patients with CAD, providing a comprehensive picture of their management. All data derived from standardized interviews, measurements and central laboratory assessments, made by centrally trained staff. To obtain a large population of women and allow comparisons between genders, we merged the EUROASPIRE IV and EUROASPIRE V surveys, which applied the same research methods and reported similar results about the secondary prevention of CAD in dysglycemic patients [4, 42].

Some limitations have to be accounted for. First, there may be a selection bias. CAD patients who were unwilling to participate were probably sicker than the participants. Therefore, it may be assumed that the present data, if anything, represent an overestimate of the quality of care, which is discouraging considering the far from optimal risk factor control reported, especially in women. Additionally, it would have been of interest to assess subsequent total mortality in relation to the quality of risk factor management and preventive treatments, but due to the rather short follow-up time the number of events was too low for total mortality to be considered as an endpoint. It may be argued that the proportion of women (approximately 25%) of all CAD cases was low although the total number of women was large (4077 of whom 2391 were dysglycaemic). We did, however, aim at recruiting a representative sample of CAD patients. With the given age restriction the proportion of women reflects the situation in the real-life clinical practice.

## Conclusions

Screening for dysglycaemia in CAD, which is still generally poorly performed across countries included in the EUROASPIRE surveys, is of special importance for women who carry a heavier burden of glucose perturbations and whose glycaemic control is poorer than in men. CV risk factor management by means of pharmacological and lifestyle interventions is significantly less well implemented in dysglycemic women with CAD, and as a consequence women with known T2DM and established CAD have a worse prognosis compared with men.

## Supplementary Information

**Additional file 1: Appendix S1.** EUROASPIRE IV & V registry: Hospital arm study centres and collaborators.

## Data Availability

Data are available from the authors upon reasonable request and with permission of EORP Oversight Committee, Executive Committee and Steering Committee of EA IV and EAV studies.

## Abbreviations

2 h-PG: 2 Hours after the glucose load
BMI: Body mass index
CV: Cardiovascular
CABG: Coronary artery bypass grafting
CAD: Coronary artery disease
DECODE: Diabetes Epidemiology: Collaborative analysis Of Diagnostic criteria in Europe
EORP: EURObservational Research Programme
EUROASPIRE: European Action on Secondary and Primary Prevention by Intervention to Reduce Events
HbA1c: Glycated haemoglobin
HDL-C: High density lipoprotein-cholesterol
HADS: Hospital Anxiety and Depression Scale
IFCC: International Federation of Clinical Chemistry and Laboratory Medicine
IFG: Impaired fasting glucose
IGT: Impaired glucose tolerance
LDL-C: Low density cholesterol- cholesterol
MI: Myocardial infarction
OGTT: Oral Glucose Tolerance test
PCI: Percutaneous coronary intervention
PG: Plasma glucose
RAAS: Renin–angiotensin–aldosterone system
T2DM: Type 2 diabetes
