# Supplementary material for: Gender differences in screening for glucose perturbations, cardiovascular risk factor management and prognosis in patients with dysglycaemia and coronary artery disease: results from the ESC-EORP EUROASPIRE surveys
Source: Cardiovasc Diabetol. 2021 Feb 11;20:38. doi: 10.1186/s12933-021-01233-6 (PMC7879645; doi:10.1186/s12933-021-01233-6)
Supplement: Supplementary file 1 — Additional file 1: Appendix S1. EUROASPIRE IV & V registry: Hospital arm study centres and collaborators. [file 12933_2021_1233_MOESM1_ESM.docx]

**Online-only text**

**EUROASPIRE IV & V registry: Hospital arm study centres and collaborators**

| **EUROASPIRE IV & V registry: Hospital arm study centres and collaborators**  **EORP Oversight Committee**  **EUROASPIRE IV&V Scientific Steering/ Expert Committees:**  **Coordinating centre**  **Diabetes centre**  **Statistical analysis centre**  **Central laboratory**  **Data management centre**  **EUROASPIRE IV Investigators**  **EUROASPIRE V Investigators**  **Table S1**  **Table S2** | **2**  **2**  **2**  **2**  **2**  **2**  **2**  **2**  **2-3**  **3-5**  **6**  **7** |
| --- | --- |
|  |  |

**EUROASPIRE IV & V registry: Hospital arm study centres and collaborators**

**EORP Oversight Committee**

Christopher P. Gale, GB (Chair); Branko Beleslin, RS; Andrzej Budaj, PL; Ovidiu Chioncel, RO; Nikolaos Dagres, DE; Nicolas Danchin, FR; David Erlinge, SE; Jonathan Emberson, GB; Michael Glikson, IL; Alastair Gray, GB; Meral Kayikcioglu, TR; Aldo P. Maggioni, IT; Klaudia Vivien Nagy, HU; Aleksandr Nedoshivin, RU; Anna-Sonia Petronio, IT; Jolien Roos-Hesselink, NL; Lars Wallentin, SE; Uwe Zeymer, DE

**EUROASPIRE IV&V Scientific Steering/ Expert Committees:**

Kornelia Kotseva, GB (Chair EUROASPIRE V Steering Committee); Guy De Backer, BE (Chair EUROASPIRE V Executive Committee); Ana Abreu, PT; Carlos Aguiar, PT; Philippe Amouyel, FR; Jolita Badariene, LT; Jan Bruthans, CZ; Almudena Castro Conde, ES; Renata Cifkova, CZ; Jim Crowley, IE; Kairat Davletov, KZ; Delphine De Smedt, BE; Dirk De Bacquer, BE; Johan De Sutter, BE; Jaap W. Deckers, NL; Mirza Dilic, BA; Vilnis Dzerve, LV; Maryna Dolzhenko, UA; Andrejs Ērglis, LV; Zlatko Fras, SI; Dan Gaita, RO; Stephan Gielen, DE; Nina Gotcheva, BG; John Goudevenos, GR; Diederick E. Grobbee, NL; Viveca Gyberg, SE; Arno W. Hoes, NL; Hosam Hasan-Ali, EG; Peter Heuschmann, DE; Piotr Jankowski, PL; Nebojsa Lalic, RS; Aleksandras Laucevicius, LT; Seppo Lehto, FI; Christos Lionis, GR; Dragan Lovic, RS; Aldo P. Maggioni, IT; Silvia Mancas, RO; Pedro Marques-Vidal, CH; Davor Miličić, HR; Erkin Mirrakhimov, KG; David Moore, IE; Evagoras Nicolaides, Cyprus; Rafael Oganov, RU; Andrzej Pajak, PL; Nana Pogosova, RU; Željko Reiner, HR; Lars Rydén, SE; Oliver Schnell, DE; Martin Stagmo, SE; Stefan Störk, DE; Jouko Sundvall, FI; Lale Tokgözoğlu, TU; Konstantinos Tsioufis, GR; Jaako Tuomilehto, FI; Dusko Vulic, BA; David A. Wood, GB (Principal Investigator)

**Coordinating centre**

Agnieszka Adamska, GB; Sabina Adamska, GB; Catriona Jennings, GB; Kornelia Kotseva, GB; David A. Wood GB

**Diabetes centre**

Viveca Gyberg, SE, Linda Mellbin, SE; Oliver Schnell, DE; Lars Rydén, SE; Jaakko Tuomilehto, FI

**Statistical analysis centre**

Dirk De Bacquer, BE; Guy De Backer, BE

**Central laboratory**

Laura Raman, FI; Jouko Sundvall, FI

**Data management centre**

EURObservational Research Programme Department, European Heart House, Sophia Antipolis, France: C. Berle, T. Ferreira, E. Fiorucci, M. Glemot, M. Konte, F. Larras, A. Maggioni, M. Manini, V. Missiamenou, C. Taylor.

**EUROASPIRE IV Investigators**

**Belgium:** *Ghent*: D. De Bacquer, G. De Backer, M. De Pauw, C. Ghysbrecht, P. Vervaet; *Ghent*: J. De Sutter, S. Pardaens, A. M Willems; *Ghent*: P. Cambier, R. Claeys, N. Deweerdt, J. Nimmegeers, H. Vandekerckhove, H. Verloove, L. Versee.

**Bosnia and Herzegovina:** *Banja Luka*: D. Vulic, D. Djekic; *Banja Luka*: G. Malesevic, S. Pejicic, S. Srdic; *Sarajevo*: M. Dilic, A. Begic, E. Hodzic, M. Kulic, N. Sabanovic-Bajramovic, E. Tahirovic; *Tuzla*: I. Iveljic, J. Kovcic, Z. Kusljugic, M. Nurkic.

**Bulgaria:** *Sofia*: N. Gotcheva, V. Baycheva, B. Georgiev, G. Vladimirov; *Sofia*: D. Gotchev, S. Ivanov.

**Croatia:** *Zagreb*: Ž. Reiner, D. Miličić , J. Samardžić; *Zagreb*: B. Perić; *Zagreb*: M. Sičaja.

**Cyprus:** *Nicosia*: E. Nicolaides, C. Eftychiou, N. Eteocleous, P. Georgiou, C. Hadjilouca, J. A. Moutiris, R. Nicolaou, K. Papadopoulos, M. Patsalou.

**Czech Republic:** *Prague*: J. Bruthans, R. Cífková, A. Krajcoviechova, P. Wohlfahrt; *Pilsen*: J. Filipovský, M. Krizek, Z. Kviderova, O. Mayer, P. Vágovičová, J. Vanek, J. Seidlerova, K. Timoracká; *Prague*: V. Adamkova, J. Belohoubek, M. Galovcova, V. Zelenkova.

**Finland:** *Kuopio*: S. Lehto, E. Kiljander, P. Kiljander, P. Kylmaoja, S. Lehto, S. Olkkonen; *Iisalmi*: J. Pennanen; Varkaus Hospital: M Herranen.

**France:** *Lille, Lomme, Tourcoing, Roubaix*: P. Amouyel, A. L. Astolfi , S. Balik, S. Beauchant , J. Dallongeville, C. Devoghelaere, N. Fievet, P. Garboni, B. Lemaire, N. Marecaux, M. Montaye

**Germany:** *Kitzinger*: W. Karmann, S. Held; *Würzburg*: P. Heuschmann, K. Eichstädt, L. Deckert, D. Fischer, A. Gerhardt, J. Kircher, Y. Memmel, K. Nolte, M. Schich, V. Wahl, M. Wagner; *Würzburg*: S. Störk, G. Ertl, S. Güntner, R. Leyh.

**Greece:** *Ioannina*: J. Goudevenos, K. Kalantzi; *Athens*: D. Athanassias, G. Goumas, P. Krimbas, D. Richter, D. Sakellariou; *Athens*: J. Agrios, I. Matthaios, E. Papadopoulou, S. Toumanidis, E. Tsouna-Hatjis; *Athens*: A. Boufidou, K. Makedou, L. Lilis.

**Ireland:** *Tallaght*: D. Moore, G. Broderick, N. Fallon, S. Storey.

**Latvia:** *Daugavpils*: I. Baronenko, G. Dormidontova, A. Dulkevica, V. Dzerve; *Riga*: A. Erglis, T. Andrejeva, N. Bricina, J. Jakovleva, A. Jaunromane, E. Keive, M. Klovane, D. Lurina, L. Makarova, D. Matisone, I. Mintale, E. Pahomova-Strautina, L. Putane, M. Stabulniece, D. Vasiljevs, G. Vevere, J. Vilks.

**Lithuania:** *Vilnius*: A. Laucevicius, I. Alitoit, J. Badariene, I. Grabliauskaite, I. Jursyte, E. Paleviciute, Z. Petrulioniene, P. Serpytis, R. Serpytis, S. Solovjova, V. Smagriunaite; *Kaunas*: R. Babarskiene, I. Ceponiene, O. Gustiene, R. Karaliute, E. Rumbinaite, R. Slapikas, V. Smalinskas, R. Verseckaite.

**Poland:** *Kraków*: A. Pająk, E. Brzezicka, R. Łysek, W. Misiowiec, R. Wolfshaut-Wolak; *Kraków*: J. Nessler; *Kraków*: P. Podolec; *Kraków*: E. Mirek-Bryniarska; *Kraków*: J. Grodecki; *Kraków*: D. Czarnecka, A. Łukaszewska, P. Jankowski; *Kraków*: P. Bogacki.

**Romania:** *Timisoara*: D. Gaita, C. Avram, E. Barzuca, L. Gaita, F. Jurca-Simina, O. C. Iancu, A. Lazar, M. Iurciuc, S. Iurciuc, M. Mal, S. Mancas, A. Mihaescu, D. Mociar, S. Mosteoru, S. Pescariu, L. Petrescu, C. Sasec, A. Schiller; *Bucharest*: L. Amarie, A. Andronic, S. Calin, A. Ciobanu, A. Cotoban, S. Guberna, L. Lungeanu, D. Mihalcea, N. Niculescu, R. Rimbas, C. Udroiu, D. Vinereanu.

**Russia:** *Moscow*: N. Pogosova, A. Ausheva, S. Boytsov, A. Kursakov, R. Oganov; *Moscow*: Y. Pozdnyakov, N. Skazin.

**Serbia:** *Nis*: D. Lovic, B. Lovic, M. Nedeljkovic, M. Ostojic; *Niska Banja*: D. Djordjevic, S. Kostic, I. Tasic; *Belgrade*: M. Zdravkovic; *Belgrade*: M. Anđić, T. Filipović, O. Ilić-Stojanović, M. Ješić-Jukić, N. Jevsnik, M. Lazović, A. Radović, D. Radović, D. Rosić, D. Spiroski, S. Stevović, T. Vidaković, V. Vuković-Dejanović.

**Slovenia:** *Ljubljana*: Z. Fras, B. Jug, A. Juhant, A. Poljancic, L. Poljancic.

**Spain:** *Madrid*: A. Castro Conde, R. Dalmau Gonzalez-Gallarza, A. M. Iniesta Manjavacas.

**Sweden:** *Malmo*: M. Stagmo, H. Jernhed, E. Stensgaard; *Stockholm*: V. Gyberg, V. Boström, C. Edman Jönsson, C. Hage.

**The Netherlands:** *Rotterdam*: J. W. Deckers, S. Khatibi F, Yongzhao; *Rotterdam*: M. Veerhoek; *Rotterdam*: P. C. Smits; *Amsterdam*: M. Minneboo, R. J. G. Peters, W. Scholte op Reimer, M. Snaterse-Zuidam.

**Turkey:** *Ankara*: L. Tokgözoğlu, S. Asil, B. Kaya, D. Koçyiğit; *Ankara*: Ç. Erol, V. Kozluca, C. Tulunay Kaya; *İzmir*: İ. Akyıldız, O. Ergene, E. Varış; *İzmir*: B. Akdeniz, Ö. Göldeli, Ö. Kozan, E. Özpelit; *Istanbul*: S. Altay, N. Çam, M. Eren; *İzmir*: M. Kayıkçıoğlu, H. Kültürsay; *Istanbul*: V. Aytekin, A. Burak Çatakoğlu; *Ankara*: A. Abacı, M. Candemir, S. Ünlü; *Istanbul*: A. Oğuz; *Ankara*: C. Barçın, S. Yaşar, M. Yokuşoğlu; *Ankara*: S. Aydoğdu, A. Temizhan, S. Ünal; *İstanbul*: H. Altuğ Çakmak, M. Çimci, Z. Öngen; *Gebze*: G. Ateş, N. Koylan; *İstanbul*: S. Emet, B. Umman; *İstanbu*l: C. Bostan, V. Sansoy; *İstanbul*: M Kemal Erol, A Kemal Kalkan; *İstanbul*: C Kaymaz, N Poçi.

**Ukraine** *Kiev*: M. Dolzhenko, T. Getman, L. Konoplyanik, L. Klimenko, L. Lobach, Y. Luchinskaya, L. Lurie, M. Lutay, E. Mitchenko, O. Nemchena, N. Nosenko, N. Perepelchenko, S. Potashev, A. Radchenko, V. Romanov, V. Shumakov, T. Simagina, Y. Sirenko, O. Sychov; *Kiev*: N. Mohnacheva, A. Verezhnikova, O. Zharinov; *Kiev*: V. Lishnevskaya, I. Mikropulo, V. Prihodko, I. Shapovalenko.

**United Kingdom:** *London*: D. Wood, A. Adamska, J. Evans, K. Ioannides, C. Jennings, A. Kasonta, K. Kotseva, H. Onyango, A. Rapacz, B. Wrotniak; *London*: S. Dubrey; *London*: M. Barbir; *London*: S. Connolly; *London*: M. Dancy; *London*: P. Collins; *London*: R. Kaprielian.

**EUROASPIRE V Investigators**

**Belgium:** *Ghent*: D. De Smedt, *Ghent*: J. De Sutter: A. M. Willems, *Ghent*: M. De Pauw, P. Vervaet, *Hasselt*: J. Bollen, E. Dekimpe, N. Mommen, G. Van Genechten, *Hasselt*: P. Dendale, *Bruxelles*: A. Bouvier, P Chenu, D. Huyberechts, A. Persu; **Bosnia & Herzegovina:** *Sarajevo*: A. Begic, M. Dilic, , A. Durak Nalbantic, A. Dzubur, N. Hadzibegic, A. Iglica, S. Kapidjic, A. Osmanagic Bico, N. Resic, N. Sabanovic Bajramovic, F. Zvizdic, *Banja Luka*:, T. Kovacevic-Preradovic, S. Popovic-Pejicic, D. Vulic, *Banja Luka*: D. Djekic, T. Gnjatic, T. Knezevic, T. Kovacevic-Preradovic, L. Kos, S. Popovic-Pejicic, B. Stanetic, G. Topic;

**Bulgaria**: *Sofia*: B. Georgiev, N. Gotcheva, A. Terziev, G. Vladimirov, *Varna*: A. Angelov, B. Kanazirev, S. Nikolaeva, D. Tonkova, M Vetkova;

**Croatia:** *Zagreb*: A. Bosnic, M. Dubravcic, M. Glavina, M. Mance, D. Milicic, S. Pavasovic, Ž. Reiner, J. Samardzic, *Zagreb*: T. Batinic, K. Crljenko, D. Delic-Brkljacic, K. Dula, K. Golubic, I. Klobucar, K. Kordic, N. Kos, M. Nedic, D. Olujic, V. Sedinic, *Zagreb*: T. Blazevic, A. Pasalic, M. Percic, J. Sikic;

**Czech Republic:** *Prague*: J. Bruthans, R. Cífková, K. Hašplová, P. Šulc, P. Wohlfahrt, *Pilsen*:, M. Cvíčela, J. Filipovský, J. Gelžinský, M Hronová, O. Mayer Jr;

**Egypt:** *Assiut:* M. Abolkassem, S. Bakery, H. B. Hamed, H. Hasan-Ali, E. Mosad, *Aswan:* M. A. Elsharef, A. Ibrahim, E. F. Kholef, *Cairo:* A. Shehata, M. Youssef*, Cairo:* E. Elhefny, H. Farid, *Zagazig*: T. M. Moustafa, M. S. Sobieh, Banha: H. Kabil, A. Abdelmordy;

**Finland:** *Kuopio and North Karelia*: E. Kiljander, P. Kiljander, H. Koukkunen, S. Lehto, J. Mustonen;

**Germany** *Halle (Saale):* C. Cremer, S. Frantz, A. Haupt, U. Hofmann, K. Ludwig, H. Melnyk, M. Noutsias, *Kitzingen:* W. Karmann; *Merseburg*: R. Prondzinsky, *Ostfildern*: C. Herdeg, T. Hövelborn, *Tübingen:* A. Daaboul, T. Geisler, T. Keller, D. Sauerbrunn, M. Walz-Ayed ; *Würzburg*: T. Ehlert, G. Ertl, P. Heuschmann , J. Käs, B. Klocke, J. Krapp, R. Leyh, S. Störk ; T. Ludwig, C. Starke, K. Ungethüm, M. Wagner, S. Wiedmann;

**Greece:** *Athens*: K. Tsioufis, P. Tolis, G. Vogiatzi, *Athens*: E. Sanidas, K. Tsakalis, *Athens*: J. Kanakakis, A. Koutsoukis*, Thessaloniki*: K. Vasileiadis, J. Zarifis, *Thessaloniki*: C. Karvounis;

**Ireland** *Galway*: J. Crowley, I. Gibson, A. Houlihan, *Galway*: C. Kelly, M. O’Donnell; **Italy:** *Cortona*: M. Bennati, F. Cosmi, B. Mariottoni, M. Morganti, *Trieste*: A. Cherubini, A. Di Lenarda, D. Radini, F. Ramani. P.O. Garibaldi-Nesima, *Catania*: M. G. Francese, M. M. Gulizia, D. Pericone;

**Kazakhstan:** *Almaty*: K. Davletov, *Aktobe*: A. Kaziyeva, B. Zholdin: *Almaty*: B. Amirov, B. Assembekov, E. Chernokurova, F. Ibragimova, A. Kodasbayev, A. Markova;

**Kyrgyzstan:** *Osh*: E. Mirrakhimov, A. Asanbaev, U. Toktomamatov, M. Tursunbaev, U. Zakirov, *Bishek*: S. Abilova, R. Arapova, E. Bektasheva, J. Esenbekova, K. Neronova, *Osh*: A. Asanbaev, K. Baigazieva, U. Toktomamatov, U. Zakirov, *Bishek*: G. Baitova, T. Zheenbekov;

**Latvia**: *Riga*: T. Andrejeva, I. Bajare, A. Erglis, G. Kucika, A. Labuce, L. Putane, M. Stabulniece. *Liepaja*: V. Dzerve, E. Klavins, I. Sime;

**Lithuania**: *Vilnius*: J. Badariene, L. Gedvilaite, D. Pečiuraite, V. Sileikienė, E. Skiauteryte, S. Solovjova, R. Sidabraite, *Kaunas:* K. Briedis, I. Ceponiene, M. Jurenas, J. Kersulis, G. Martinkute, A. Vaitiekiene, K. Vasiljevaite, R. Veisaite, *Siauliai*: J. Plisienė, V. Šiurkaitė, Ž. Vaičiulis;

**Poland**: *Krakow*: D. Czarnecka, P. Jankowski, P. Kozieł, *Krakow*: P. Podolec, *Krakow*: J. Nessler, *Krakow*: P. Gomuła, *Krakow*: E Mirek-Bryniarska, *Krakow*: P. Bogacki, *Krakow*: A Wiśniewski, *Krakow*: A. Pająk, R. Wolfshaut-Wolak, *Białystok*: J. Bućko, K. Kamiński, M. Łapińska, M. Paniczko, A. Raczkowski, E. Sawicka, Z. Stachurska, M. Szpakowicz, *Białystok*: W. Musiał, *Białystok*: S. Dobrzycki, *Białystok*: J. Bychowski, *Warsaw*: D. A. Kosior, A. Krzykwa, M. Setny, *Warsaw*: D. A. Kosior, *Józefów*: A. Rak, *Katowice*: Z. Gąsior, M. Haberka, *Katowice*: Z. Gąsior, M. Haberka, K. Szostak-Janiak, *Jaworzno*: M. Finik, J. Liszka;

**Portugal:** *Coimbra :* A. Botelho, M. Cachulo, J. Sousa, A. Pais, *Lisboa*: C. Aguiar, A. Durazzo, D. Matos, R. Gouveia, G. Rodrigues, C. Strong. *Evora:* R. Guerreiro, J. Aguiar, *Lisboa*: A. Abreu, M. Cruz, P. Daniel, L. Morais, R. Moreira, I. Rodrigues, F. Silva, M. Selas;

**Romania**: *Timisoara:* A. Apostu, O. Cosor, D. Gaita, L. Gaita, L. Giurgiu, C. Hudrea, S. Mancas, D. Maximov, B. Moldovan, S. Mosteoru, R. Pleava, *Constanta*: M. Ionescu, I. Parepa;

**Russian Federation**, *Moscow*: A Arutyunov, A. Ausheva, S. Isakova, A. Karpova, N. Pogosova A. Salbieva, O. Sokolova, A. Vasilevsky, *Moscow*: Y. Pozdnyakov, *Baranul*: O. Antropova, L. Borisova, I. Osipova;

**Serbia**: *Nis*: D Lovic, *Belgrade*: M. Aleksic, B. Crnokrak, J. Djokic, S. Hinic, T. Vukasin, M. Zdravkovic, *Belgrade*: A. Jotic, N. M. Lalic, K. Lalic, L. Lukic, T. Milicic, M. Macesic, J. Stanarcic Gajovic, M. Stoiljkovic, *Nis*: D. Djordjevic, S. Kostic, I. Tasic, A. Vukovic;

**Slovenia**: *Ljubljana*: Z. Fras, B. Jug, A. Juhant, A. Krt, U. Kugonjič;

**Spain**: *Caceres*: D. Chipayo Gonzales, J. J. Gómez Barrado, Z. Kounka, G. Marcos Gómez, M. V. Mogollón Jiménez, C. Ortiz Cortés, P. Perez Espejo, Y. Porras Ramos, *Cadiz*: R. Colman, J. Delgado, E. Otero, A. Pérez, *Jaén*: M. R. Fernández-Olmo, J. Torres-LLergo, C. Vasco, *Madrid*: E. Barreñada, J. Botas, R. Campuzano, Y. González, M. Rodrigo, *Madrid*: C. de Pablo, E. Velasco, S. Hernández, C. Lozano, P. González, *Madrid:* A. Castro, R. Dalmau, D. Hernández, F. J. Irazusta, A. Vélez, C. Vindel, *Malaga*: J. J. Gómez-Doblas, V. García Ruíz, L. Gómez. M. Gómez García, M. Jiménez-Navarro, A. Molina Ramos, *Mérida:* D. Marzal, G. Martínez, R. Lavado, A. Vidal;

**Sweden:** *Stockholm*: V. Boström-Nilsson, B. Kjellström, L. Rydén, B. Shahim, S. Smetana, *Malmö*: O. Hansen, E. Stensgaard-Nake;

**The Netherlands**: *Rotterdam*: J. W. Deckers , A. J. Klijn, T. J. P. Mangus, *Amsterdam*: R. J.G. Peters, W. Scholte op Reimer, M. Snaterse;

**Turkey**: *Ankara*: S. Aydoğdu, Ankara: Ç. Erol, S. Oztürk, C. Tulunay Kaya, *Izmir*: Y. Ahmetoğlu, O. Ergene, *Izmir :* B. Akdeniz, D. Çırgamış, *Izmir* : S. Akkoyun, H. Kültürsay, M. Kayıkçıoğlu, *Istanbul*: A. B. Çatakoğlu, *Ankara:* A. Çengel, A. Koçak, *Istanbul*: M. A. Ağırbaşlı, G. Açıksarı, M. E. Çekin, *Ankara*: E. B. Kaya, D. Koçyiğit, L. Tokgözoğlu, *Istanbul:* Z. Öngen, E. Özmen, *Istanbul*: V. Sansoy, A. Kaya, V. Oktay, *Ankara*: A. Temizhan, S. Ünal, İ. Yakut, *Istanbul*: A. K. Kalkan, *Ankara*: E. Bozkurt, H. A. Kasapkara;

**Ukraine:** *Kiev*: M. Dolzhenko, C. Faradzh, L. Hrubyak, L. Konoplianyk, N. Kozhuharyova, L. Lobach, V. Nesukai, O. Nudchenko, T. Simagina, L. Yakovenko, *Kryvoy Rig*: V. Azarenko, V. Potabashny, *Lviv*: A. Bazylevych, M. Bazylevych, K. Kaminska, *Zaporizhzhya*: L. Panchenko, O. Shershnyova, *Kharkiv* : T. Ovrakh, S. Serik; *Dnipro*: T. Kolesnik, H. Kosova;

**United Kingdom**: *London*: A. Adamska, S. Adamska, C. Jennings, K. Kotseva, D. Wood, *Hull*: A. Hoye, P. Atkin, D. Fellowes, *Bradford*: S. Lindsay, C. Atkinson, C. Kranilla, M. Vinod, London: D. Wood, H. Abbass, N. Rhoualmi, *Harrogate*: Y. Beerachee, C. Bennett, M. Broome, A. Bwalya, L. Caygill, L. Dinning, A. Gillespie, R. Goodfellow, J. Guy, T. Idress, C. Mills, C. Morgan, N. Oustance, N. Singh, M. Yare, *Hinchingbrooke*: J. M. Jagoda; H. Bowyer, V. Christenssen, A. Groves, *Sutton in Ashfield*: A. Jan, A. Riaz, M. Gill, T. A. Sewell, *Stevenage:* D. Gorog, M. Baker, P. De Sousa, T. Mazenenga, *Peterborough*: J. Porter, F. Haines, T. Peachey, J. Taaffe, K. Wells, *North Shields*: D. P. Ripley, H. Forward, H. McKie, S. L. Pick, H. E. Thomas, *Wakefield*: P. D. Batin, D. Exley, T. Rank, J. Wright. Milton Keynes: A. Kardos, S-B. Sutherland, L. Wren, *Oxford*: P. Leeson, D. Barker, B. Moreby, J. Sawyer, *Reading*: J. Stirrup*, M. Brunton, *Morecambe Bay*: A. Brodison, J. Craig, S. Peters. *West Middlesex:* R. Kaprielian, A. Bucaj, K. Mahay, M. Oblak, *York:* C. Gale, M. Pye, Y. McGill, H. Redfearn, M. Fearnley

**Table S1**: WHO definition of glycemic state. ^1^

|  | **Venous plasma glucose (mmol/L)** | |
| --- | --- | --- |
|  | **Fasting** | **2-hour post-load** |
| **Normoglycaemic** | <6.1 | <7.8 |
| **Impaired fasting glucose (IFG)** | 6.1 – 6.9 | < 7.8 |
| **Impaired glucose tolerance (IGT)** | <7.0 | 7.8–11.0 |
| **Diabetes** | ≥7.0 | >11.0 |

1. World Health Organization. Definition and diagnosis of diabetes mellitus and intermediate hyperglycaemia: report of a WHO/IDF consultation [Internet]. WHO. Available from https://www.who.int/diabetes/publications/diagnosis_diabetes2006/en/. 2020.

**Table S2**: number of endpoint events in participants with newly detected dysglycaemia and with previously known diabetes, divided by gender.

|  | **Newly detected dysglycaemia** | | **Previously known diabetes** | |
| --- | --- | --- | --- | --- |
|  | **Men**  N=2,812 | **Women**  N=980 | **Men**  N=3,188 | **Women**  N=1,222 |
| **Total Endpoint** | 340 | 105 | 500 | 233 |
| **Fatal CVD**  **PCI**  **CABG**  **Acute MI**  **Stroke**  **Heart failure** | 30  169  14  59  45  86 | 7  44  4  14  11  37 | 50  212  34  85  70  150 | 20  81  15  40  31  90 |

CABG: coronary artery bypass grafting; CVD: cardiovascular disease; MI: myocardial infarction; PCI: percutaneous coronary intervention.
